# scFed: federated learning for cell type classification with scRNA-seq

**DOI:** 10.1093/bib/bbad507

**Published:** 2024-01-13

**Authors:** Shuang Wang, Bochen Shen, Lanting Guo, Mengqi Shang, Jinze Liu, Qi Sun, Bairong Shen

**Affiliations:** Joint Laboratory of Artificial Intelligence for Critical Care Medicine, Department of Critical Care Medicine and Institutes for Systems Genetics, Frontiers Science Center for Disease-related Molecular Network, West China Hospital, Sichuan University, 610212, Chengdu, China; Department of Bioinformatics, Hangzhou Nuowei Information Technology Co., Ltd, 310053, Hangzhou, China; Department of Bioinformatics, Hangzhou Nuowei Information Technology Co., Ltd, 310053, Hangzhou, China; Department of Bioinformatics, Hangzhou Nuowei Information Technology Co., Ltd, 310053, Hangzhou, China; Department of Bioinformatics, Hangzhou Nuowei Information Technology Co., Ltd, 310053, Hangzhou, China; Department of Biostatistics, Virginia Commonwealth University, 23298, Richmond, VA, USA; Department of Bioinformatics, Hangzhou Nuowei Information Technology Co., Ltd, 310053, Hangzhou, China; Joint Laboratory of Artificial Intelligence for Critical Care Medicine, Department of Critical Care Medicine and Institutes for Systems Genetics, Frontiers Science Center for Disease-related Molecular Network, West China Hospital, Sichuan University, 610212, Chengdu, China

**Keywords:** federated learning, scRNA-seq, classification, cell type

## Abstract

The advent of single-cell RNA sequencing (scRNA-seq) has revolutionized our understanding of cellular heterogeneity and complexity in biological tissues. However, the nature of large, sparse scRNA-seq datasets and privacy regulations present challenges for efficient cell identification. Federated learning provides a solution, allowing efficient and private data use. Here, we introduce scFed, a unified federated learning framework that allows for benchmarking of four classification algorithms without violating data privacy, including single-cell-specific and general-purpose classifiers. We evaluated scFed using eight publicly available scRNA-seq datasets with diverse sizes, species and technologies, assessing its performance via intra-dataset and inter-dataset experimental setups. We find that scFed performs well on a variety of datasets with competitive accuracy to centralized models. Though Transformer-based model excels in centralized training, its performance slightly lags behind single-cell-specific model within the scFed framework, coupled with a notable time complexity concern. Our study not only helps select suitable cell identification methods but also highlights federated learning’s potential for privacy-preserving, collaborative biomedical research.

## INTRODUCTION

Single-cell RNA sequencing (scRNA-seq) associates gene expression data with an individual cell in a sample. Cellular heterogeneity in RNA transcripts is critical to answer questions for disease development and treatment. Therefore, it is no surprise of the growing enthusiasm to explore the unique transcriptomic profile of each cell by scRNA-seq for cell type identification.

Unlike bulk RNA-seq, the expression of genes from scRNA-seq is highly sparse due to limited sequencing depth per cell, which increases the chance of model overfitting and hinders downstream analysis [1]. The observed zeros can either be a true gene expression level or they are the result of methodological noise. Furthermore, gene expression data measured via transcriptomic profiling is high-dimensional profile data. Compared with relatively small sample size, the high-dimensional gene expression from scRNA-seq is subject to the curse of dimensionality. The simplest and effective way to deal with data sparsity and the curse of dimensionality is to increase the sample size [2], which requires access to a large amount of diverse datasets.

Although aggregating single-cell gene expression datasets can bolster sensitivity and robustness of cell type identifications [3], the adoption of dataset aggregation is hampered by privacy regulations since it is often restricted by them. As highlighted by Ferguson [4], the field of bioinformatics, which includes scRNA-seq data, is fraught with ethical and privacy concerns. The potential exists for individuals to be identified through their genetic data, and sharing and transferring personal genetic data might lead to possible exposure of sensitive health information. Therefore, rigorous regulations, such as General Data Protection Regulation (GDPR) and Health Insurance Portability and Accountability Act (HIPAA), have been developed to regulate the process of accessing and analyzing such data. Given these constraints, privacy-preserving federated learning solutions play a vital role in helping researchers to aggregate and explore single-cell gene expression datasets without sharing the original data of each institution.

To collaboratively learn a shared cell type identification model while keeping all the training data locally, a growing number of federated learning approaches are being adapted to automatically label cells in scRNA-seq experiments. PriCell was proposed as a federated neural network learning approach for disease-associated cell classification [5]. scPrivacy utilized federated deep metric learning algorithms to train the federated cell type identification model on multiple institutional datasets in data privacy protection manner [6]. PPML-Omics [7] analyzed data from three sequencing technologies with a privacy-preserving federated framework, clustering cell populations with Auto-encoder and k-means clustering. Those approaches align with the growing emphasis on ensuring data privacy in bioinformatics, as underscored by the challenges and concerns presented in the field. Federated-learning-based scRNA-seq classification methods are relatively new compared with traditional scRNA-seq classification methods, but they still have a common goal to accurately annotate cells without private information leakage. Various machine learning approaches, such as neural network, support vector machine (SVM) and gradient boosting machine, have been utilized to identify cell types [8]. Moreover, Transformer-based models have emerged as an additional choice for cell type identification, leveraging their self-attention mechanisms to capture complex cellular patterns [9, 10]. While UniFed demonstrated that the selection of classification methods is the main factor that affected model performance in federated learning frameworks [11], the absence of a detailed comparative study of classification methods in federated learning for scRNA-seq leaves users without clear guidelines to select the optimal method tailored to their specific challenges within this framework.

Here, we propose scFed as a unified federated learning framework to benchmark a range of classification methods, providing researchers with a systematic guide to conduct scRNA-seq analysis while ensuring data privacy. Our study employed both general-purpose and single-cell-specific classifiers for cell type identification with scRNA-seq. While SVM, and XGBoost served as general-purpose classifiers, ACTINN [12] tailored specifically for scRNA-seq data. The selection of SVM and XGBoost was based on their established efficacy across a range of datasets [8], whereas ACTINN was incorporated for its expertise in scRNA-seq data. Eight publicly available scRNA-seq of different sizes, species and technologies were employed for performance comparison. The performance of federated-learning-based classification methods was evaluated based on their accuracy and computation time. We performed several experiments covering different aspects of federated learning and classification tasks, such as datasets, client numbers and algorithm comparison. We also integrated Geneformer [10], a Transformer-based model, into the scFed framework to evaluate its potential for cell type identification. While it exhibited promising classification capabilities, it also posed significant computational demand. Thus, we benchmarked its performance against other classification methods, focusing on both accuracy and computation time, to provide a comprehensive view for this task. Our experiments revealed considerable variations in classification performance and computation time across different classification algorithms and our assessment demonstrated scFed’s effectiveness, time efficiency and robustness for privacy-preserving integration of multiple client datasets.

## METHODS

To quantitatively evaluate the federated learning framework for single cell classification, we propose scFed to integrate several single cell classification algorithms to the federated learning framework.

### System overview

**Figure 1:**
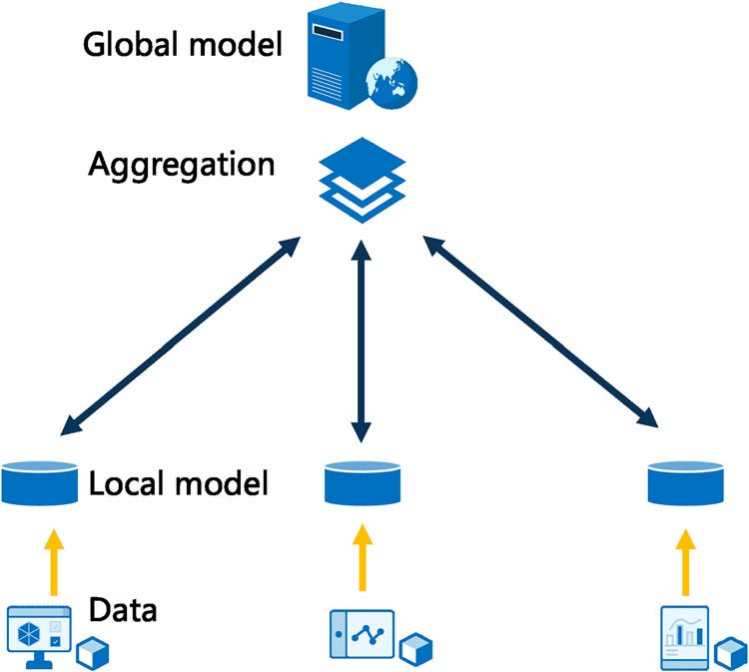
The workflow of scFed: clients use local gene expression data from scRNA-seq to train local models; the local models are used to update the global model. The aggregated global model is passed to the local models for further training.

We summarize the workflow of scFed’s system as shown in Figure 1. It is a federated learning framework that allows a global model to be trained using decentralized data scattered among a large number of different clients, without uploading client data to servers. Essentially, the framework assumes the existence of $N$ activated clients, each possessing their own dataset denoted by $D_k$. Our objective is to develop a cell type identification model that incorporates the datasets from $N$ clients.

### Classification algorithm

Prioritizing data privacy through the adoption of the federated learning framework, this workflow supports four key classification algorithms: neural networks, tree-based models, SVMs and Transformer-based models, which are also fundamental algorithms for single cell classification. In this section, we will discuss the federated-learning-based classification algorithms in detail.

#### Neural network (ACTINN)

With the strong ability of learning high level features from data, deep learning networks do not need the domain knowledge to select features, which is beneficial for the classification of a huge number of cells. A variety of deep learning models have been explored to identify cell types [13]. A recent cell classification method, ACTINN [12], employs a fully connected neural network for cell type classification. In this study, we integrate ACTINN to scFed by training neural network models with a variant of FedAvg algorithm [14] as shown in Algorithm 1.


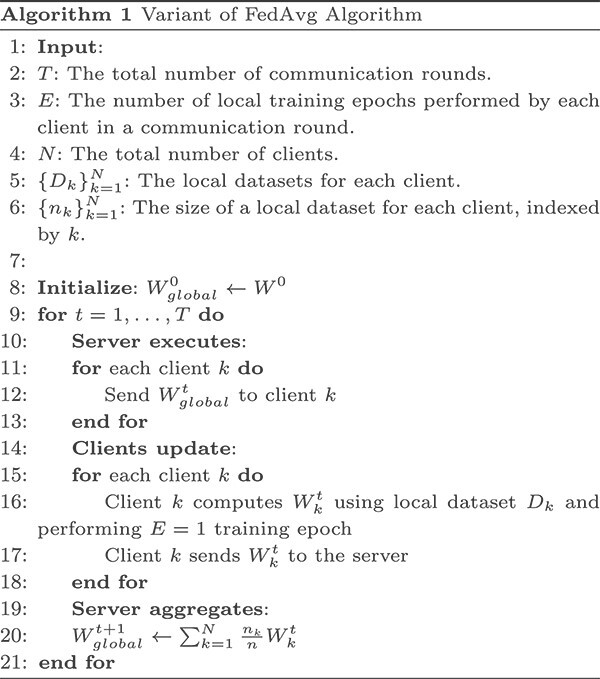


The variant of FedAvg algorithm, as presented in Algorithm 1, is a federated learning approach designed for efficient communication in deep learning networks with decentralized data. The algorithm begins by initializing the global model weights ($W^0_{global}$) and iterating through $T$ communication rounds. In the original FedAvg algorithm, each client performs $E$ epochs of local stochastic gradient updates on their local data before sending an updated model to the server. By setting $E$ to 1, each client only performs a single pass over their data, which is the same to the one weight updated round in a centralized setting, thus making it more comparable with a centralized training process for scFed’s performance evaluation. During each round, the server selects all clients to participate in computation. The server then sends the global model weights ($W^t_{global}$) to the clients. Each client fine-tunes its local model by performing a training epoch over its local dataset ($D_k$) and computes the updated model weights ($W^t_k$). Once completed, the clients send their updated weights back to the server. Finally, the server aggregates the updated weights from all clients, updating the global model weights ($W^{t+1}_{global}$) based on the weighted sum of the local weights, considering the relative size of each client’s dataset. This process is repeated for a predefined number of communication rounds, ultimately converging to an effective model that has learned from the decentralized data.

#### Support vector machine

SVMs [15] have gained significant popularity as a classification method in recent years, owing to its strong theoretical foundation, high accuracy and robustness to overfitting. SVM has been extensively applied to classify gene expression data measured on scRNA-seq data [16], addressing the challenges of high dimensionality, sparsity and noise inherent in such data.

In the context of federated learning, constructing a federated SVM with mathematical rigor is essential to ensure effective collaboration and privacy preservation. For this purpose, we specifically focus on the linear SVM, as it provides a more straightforward and computationally efficient approach compared with nonlinear kernels while still offering satisfactory performance. The linear SVM model can be represented as the coefficient for the orthogonal vector to the hyperplane and the intercept of this vector, which define the decision boundary for classification.

In a federated learning setup, each participating client holds a local dataset and trains a linear SVM model independently, without sharing the raw data. The locally trained models, consisting of weight vectors and intercepts, can then be aggregated and converted into a global model [17]. This is achieved by computing a weighted average of the local models with the weights determined by the relative size of each client’s dataset. The resulting global model captures the collective knowledge from all participating clients while preserving data privacy.

#### Tree-based model (XGBoost)

Both single decision tree and ensemble decision tree models, such as gradient boosting decision trees (GBDT) [18] and random forests, could be learned via federated learning. Owing to GBDT’s excellent performance in classification applications, it has been widely used for single cell classification [19]. In this work, we train an XGBoost model via a federated learning framework to avoid leak of client data privacy. The framework of federated XGBoost training is implemented with four steps in each communication. Firstly, the server sends the initial parameters or the new tree to the clients. Secondly, the clients update the gradient histogram separately. Thirdly, the clients send the gradient histogram to the server. Finally, the server merges the histogram and boosts a new tree [20].

#### Transformer-based model (Geneformer)

Transformer-based models are capable of analyzing vast datasets of single-cell transcriptomic data [9, 10]. Its capability to capture long-range dependencies in data points makes it as an effective tool for exploring biological information in computational contexts. A Transformer-based model called Geneformer [10] is pretrained on a large corpus of around 30 million single-cell transcriptomes to facilitate context-specific predictions. Utilizing Geneformer for classification tasks, it is necessary to fine-tune it with a specific dataset. Within scFed, the fine-tuning of Geneformer primarily comprises the following four steps. Firstly, each client loads the pretrained model. Secondly, clients conduct local training to fine-tune the parameters. Thirdly, each client sends the model parameters of this round of fine-tuning to server. Finally, the server aggregates and disseminates the model parameters to all the clients.

## RESULTS

We evaluated our proposed scFed in terms of model accuracy, scalability with the number of clients, classification algorithm feasibility and runtime analysis. The generalizability of our federated learning workflow was tested through both intra-dataset and inter-dataset classifications. Given the computational intensity of Transformer architectures, we conducted a specialized evaluation of Geneformer. Using the Zhengsorted dataset as a benchmark, we evaluated Geneformer with both classification accuracy and runtime metrics.

### Datasets

A total of eight scRNA-seq datasets were used to evaluate and benchmark scFed with all classification methods, from which all datasets were used for intra-dataset evaluation, and five datasets were used for inter-dataset evaluation. Datasets vary across sequencing protocols, species and tissue (Table 1). Five pancreas datasets sequenced with different sequencing protocols were used. BaronHuman [21], Muraro [22], Segerstolpe [23] and Xin [24] datasets are all from the human pancreas, and BaronMouse [21] is from mouse pancreas. Zhengsorted [25] are sequenced from human peripheral blood mononuclear cells. AMB dataset [26] is from the allen mouse brain. The Tabula Muris (TM) dataset represents relatively large scRNA-seq dataset (>50 000 cells)[27]. More details about datasets were described in [13].

**Table 1:** Overview of datasets used in the evaluation and benchmarking of scFed

**Dataset**	**#cells**	**#cell types**	**Protocol**
AMB	12 832	4	SMART-Seq v4
BaronHuman	8569	14	inDrop
BaronMouse	1886	13	inDrop
Muraro	2122	9	CEL-Seq2
Segerstolpe	2133	13	SMART-Seq2
Xin	1449	4	SMARTer
TM	54 865	55	SMART-Seq2
Zhengsorted	20 000	10	10X CHROMIUM

In our intra-dataset classification, we randomly split the entire dataset into 80% as training data and 20% as test, and evenly distribute the training set over $N$ clients. We used the same training and test datasets for each set of comparative experiments. In our inter-dataset classification study, first, we combined the four human pancreas datasets (Xin, BaronHuman, Muraro and Segerstolpe) and then used three of them as the training dataset and the remaining one as the test dataset. In this case, each client holds a training dataset, and they are integrated to train scFed. For data preprocessing, we filtered genes with zero counts across all cells. To remove the influence of technical effects while preserving true biological heterogeneity, a CPM [28] normalization was applied for count depth scaling. Next, the data of gene expression were log-transformed using log2(count + 1).

### Implementation

The FL framework is implemented in python and utilizes socket programming to establish connections between server and clients, thereby maintaining privacy and efficient use of distributed data. The system is powered by two Intel(R) Xeon(R) Platinum 8358 CPUs running at 2.60GHz with 128 threads on 64 cores and 512 GB RAM. In Geneformer setting, the experiments were equipped with two Hygon C86 CPUs at 2.00 GHz with 128 threads on 64 cores, 128 GB RAM and NVIDIA Tesla A40 GPU.

We use the TensorFlow library to construct the federated version of ACTINN. The structure of the global neural network model is saved and replicated across clients by a federated server. Subsequently, model parameters are extracted from the local models and sent to the server for aggregation. For neural-network-based algorithm ACTINN, we used initial learning rates of 1e-4 with adam optimization for each client, and a mini-batch of size 128 was sampled from the dataset.

We choose the scikit-learn library, which provides comprehensive tools and functions for SVM models, to access the weights of an SVM model and perform the model aggregation while ensuring the preservation of data privacy across all clients. Linear SVM is configurated by SGDClassifier in scikit-learn with hinge loss function.

For XGBoost model, we use XGBoost [18], an open-source software library, to implement federated XGBoost. Federated XGBoost is a gradient boosting library for the federated setting which enables multiple parties to jointly compute a model while keeping their data on site, avoiding the need for a central data storage. Analogous to communication rounds, we set the number of rounds for boosting to 30.

For the implementation of Transformer-based model in scFed, we use Geneformer [10] to implement federated Geneformer. Leveraging the pretrained weights from Geneformer, we perform fine-tuning by appending a task-specific transformer layer and use the trainer provided by the Huggingface Transformers library [29]. Consistent hyperparameters are applied for fine-tuning: max learning rate $5 \times 10^{-5}$, linear scheduler with warmup, Adam optimizer with weight decay fix, warmup steps 500, weight decay 0.001 and batch size 4.

### Statistical analysis

We conducted a Wilcoxon signed-rank test, which tested if there was significant difference in accuracy between the two models. By assessing the *P*-value of the Wilcoxon statistical test, we were able to establish whether the null hypothesis (i.e. no significant difference between the global and centralized models) could be rejected in favor of the alternative hypothesis (a significant difference between the two models). A *P*-value greater than 0.05 indicated that we lacked sufficient evidence to reject the null hypothesis. In the following reports, the *P*-values for comparison between the corresponding boxplots are shown as an interval. The thresholds are represented in a ‘start’ format as [1e-4, ‘****’], [1e-3, ‘***’], [1e-2, ‘**’], [0.05, ‘*’], [1, ‘ns’].

### Benchmarking federated learning for single cell classification (Intra-dataset evaluation)

In this experiment, we evaluated the scFed performance by training and testing subsets of cells included in the same scRNA-seq data. We named this an intra-dataset evaluation. The comparisons were made by reporting results from the following scenarios: (a) A centralized training data model for cell type identification, which served as our baseline. (b) Individual client trains its own local model without collaboration, with the average F1-score of all local models reported. (c) scFed is applied to obtain global models for cell type identification. For all experiments, we maintain a fixed test datasets to assess the centralized, local and global model performance with five independent repetitions performed to determine the classification results.

#### Performance evaluation across different datasets

**Figure 2:**
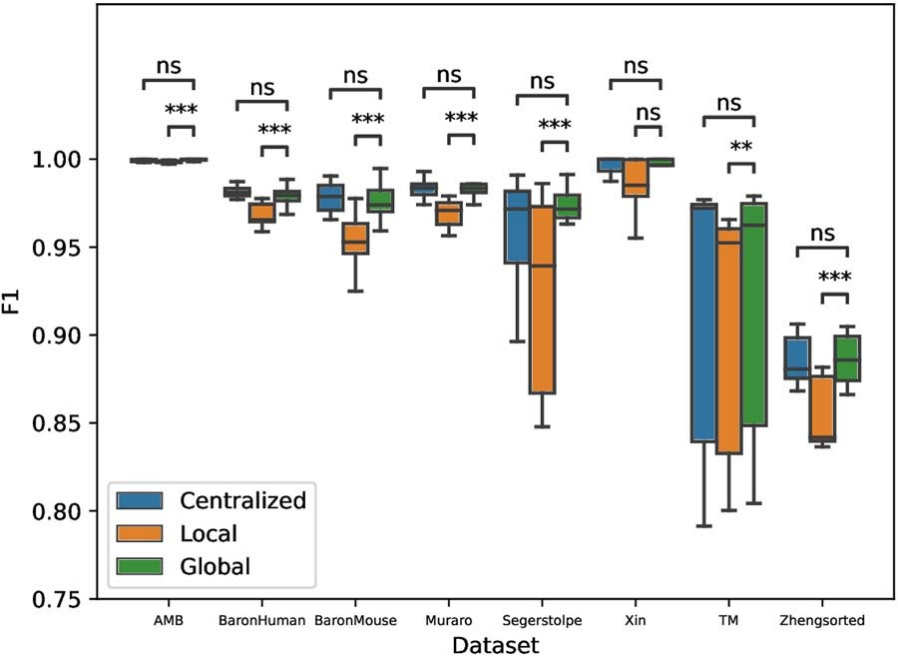
Comparison of centrialized, local and global model for intra-dataset cell type classification performance among eight datasets. The F1 scores are recorded after five independent repetitions. The *P*-value intervals shown at the top of the figure are calculated with a Wilcoxon signed-rank test for the comparison between the corresponding boxplots.

We utilized eight datasets over $N=5$ clients to assess the performance of global models implemented via scFed in comparison with local and centralized models for cell type identification tasks. Each boxplots in Figure 2 collectively displayed the F1 scores of cell type classification with algorithms of SVM, ACTINN and XGBoost.

Drawing from the Wilcoxon signed-rank test results, as illustrated in Figure 2, we observed no significant statistical difference in performance between our global models, trained using scFed, and traditional centralized models across all evaluated datasets. This demonstrates that scFed’s global model not only matches the performance of the centralized model but does so while maintaining crucial data privacy considerations. It also suggests that scFed can be a viable alternative for single-cell classification tasks while maintaining data privacy. Furthermore, global models outperform local models except for Xin dataset, which is the smallest one in our study. This indicates that federated learning can effectively aggregate and learn from the information distributed across multiple clients, leading to a more accurate and robust global model.

#### Performance evaluation across different classification algorithms

**Figure 3:**
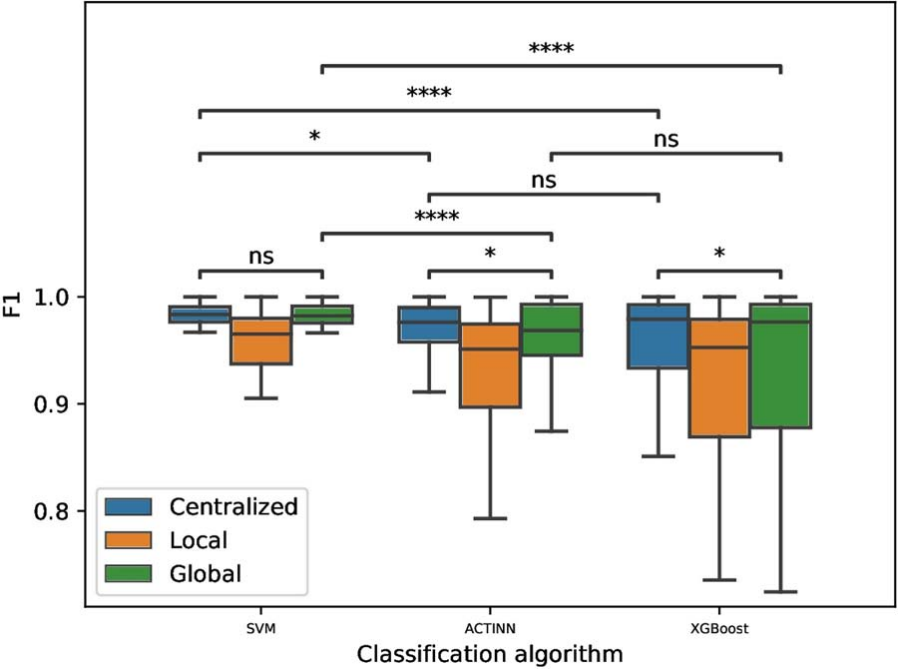
Comparison of centralized, local and global model for intra-dataset cell type classification performance among SVM, ACTINN and XGBoost algorithms. The F1 scores are recorded after five independent repetitions. The *P*-value intervals shown at the top of the figure are calculated with a Wilcoxon signed-rank test for the comparison between the corresponding boxplots.

In this experiment, we also conducted thorough comparisons of centralized, local and global models to understand the effect of classification algorithms on the cell type identification performance. Each boxplots in Figure 3 collectively displayed the F1 scores of cell type classification for eight datasets as shown in Table 1. In this set of experiments, we still fixed the client number to be five.

Figure 3 shows that the highest performance for cell type classification is attained by the centralized SVM model. The global SVM model demonstrates a performance level statistically indistinguishable from that of the centralized SVM model. Both global SVM model and centralized SVM model outperfoms corresponding ACTINN and XGBoost models, whose statistical significance was confirmed using a Wilcoxon signed-rank test. In all cases, global models surpass their local counterparts, regardless of the classification algorithm employed. When comparing ACTINN and XGBoost, no significant performance differences were observed between their centralized and global models. This finding highlights that both algorithms provide comparable results in the context of scRNA-seq cell type classification.

#### Performance evaluation over different numbers of clients

**Figure 4:**
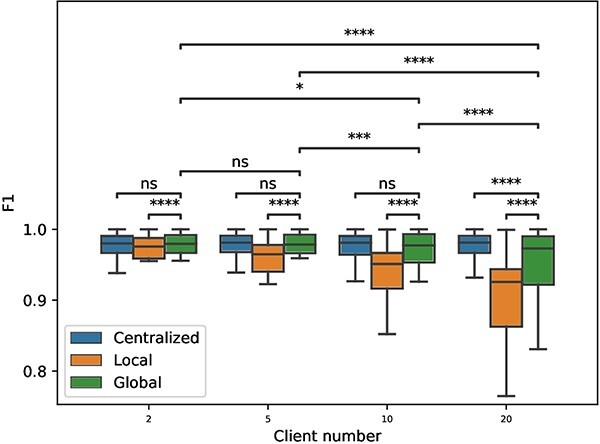
Comparison of centralized, local and global model for intra-dataset cell type classification performance varying numbers of clients. The F1 scores are recorded after five independent repetitions. The *P*-value intervals shown at the top of the figure are calculated with a Wilcoxon signed-rank test for the comparison between the corresponding boxplots.

In this section, we investigated the impact of varying the number of clients on the performance of scFed. We systematically varied the number of clients participating in the federated learning process among 2, 5, 10 and 20. Figure 4 shows the scalability of scFed with the number of clients. Each boxplots collectively displayed the F1 scores of cell type classification with algorithms of SVM, ACTINN and XGBoost gathering from eight datasets as shown in Table 1.

Figure 4 demonstrates that scFed yields comparable performance with centralized models and significantly surpasses local models in most client number setting of this experiment, except for $N=20$. When evaluating classification performance across various client quantities within scFed, we noticed no substantial difference between $N=2$ and $N=5$. However, a rise in client numbers to 20 resulted in a drop in classification performance for both global and local models. According to the results of the Wilcoxon signed-rank test, significant differences are apparent among global models as client numbers increase, with the exception of the comparison between $N=2$ and $N=5$.

### Performance evaluation across datasets (Inter-dataset evaluation)

In order to examine the generalization of the proposed model, we evaluated the performance of cross-dataset classification, which is a more realistic scenario. Since the Xin, BaronHuman, Muraro and Segerstolpe datasets are all from the human pancreas, we used these four datasets for the validation. Common cell types among these four datasets are alpha, beta, delta and gamma, so we extracted the four cell types from each dataset for combination. Five independent repetitions were performed to determine the classification results.

Before combining the datasets, we preprocessed the data using CPM normalization and log-transformation. We then standardized each dataset by min–max scaling to make the four datasets on the same level. We conducted four experiments. In each experiment, three of the four datasets were used as the training dataset and one was left as the test dataset. This approach allowed us to assess the model’s generalization capabilities across different datasets while maintaining a consistent evaluation setup.

**Figure 5:**
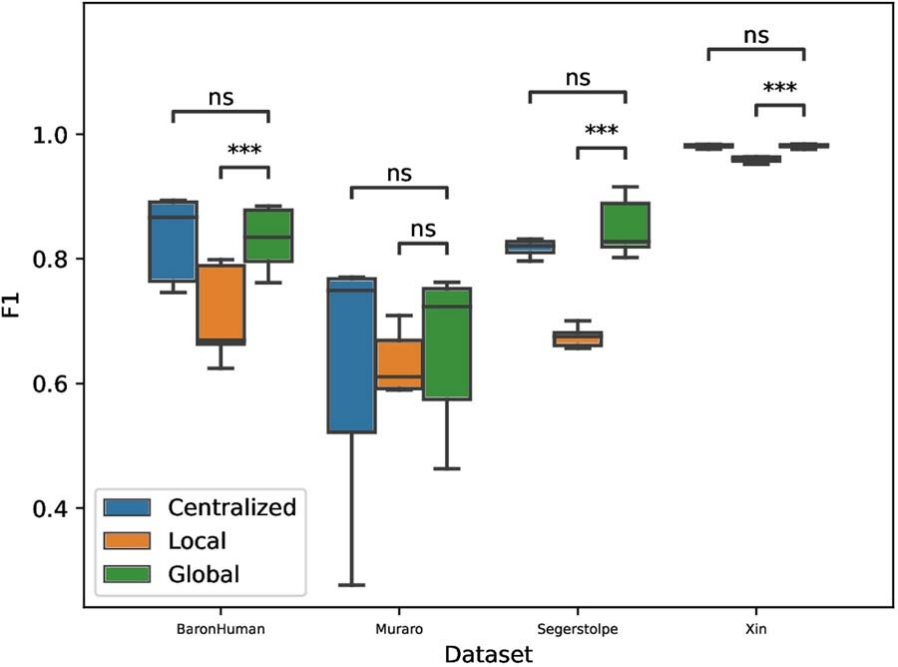
Comparison of centralized, local and global model for inter-dataset cell type classification performance taking ‘BaronHuman’, ‘Muraro’, ‘Segerstolpe’ and ‘Xin’ as the test dataset, respectively. The F1 scores are recorded after five independent repetitions. The *P*-value intervals shown at the top of the figure are calculated with a Wilcoxon signed-rank test for the comparison between the corresponding boxplots.

Figure 5 revealed no significant statistical difference between the centralized and global models’ performances, as evidenced by the Wilcoxon signed-rank test results. This lack of difference underscores the effectiveness of scFed as a model that can perform comparably with centralized models without compromising the privacy of local original data. However, a striking improvement was observed when comparing the global models with local ones. The global model, which integrated information from multiple datasets, demonstrated significantly superior performance in comparison with local models. This enhancement indicates the power of scFed to harness shared information across multiple clients, thereby enhancing the performance in a real-world, heterogeneous data scenario. These results substantiate the scFed model’s potential as a valuable tool in federated learning, capable of effectively generalizing across diverse datasets.

### Runtime comparison

In this section, we provided a comprehensive analysis of the runtime performance of the SVM and ACTINN models, which we have specifically developed for the federated learning framework, referred to as the global model (scFed). This evaluation also includes comparisons with their local and centralized counterparts. Please note that this study primarily emphasizes the models we have tailored and implemented for the federated learning environment, thus we have chosen not to include a runtime comparison for the federated XGBoost model, which is an externally sourced implementation.

To make a fair runtime comparison among global, centralized and local models, we standardized training iterations for comparative pipelines. Specifically, we fixed the number of training iterations to 100. In the case of the global model (scFed), 100 communication rounds were performed, with each round iterating over the entire dataset once. For the centralized and local models, they iterated over the training dataset 100 times during the training process.

We performed five independent runs employing the Zhengsorted dataset for the global, centralized and local model, and noted the training time. We reported the average of those five training times in Table 2. We observed that the global training time for the SVM models, varying the number of clients from 2, 5, 10 to 20, is approximately two to three times longer than the centralized training time. The local training time was slightly longer than the centralized one. In the case of ACTINN, as shown in Table 2, the global training time in the scFed framework exhibits a 3-fold increase relative to the centralized model, excepting for the scenario with 20 clients. The time difference between the centralized and local models for ACTINN was negligible. This analysis underscores the computational performance of scFed in cell type classification tasks, illuminating its computational efficiency is in an accessible range, especially considering the scale and complexity of the task.

**Table 2:** Training execution time comparison of centralized, local and global scenarios for cell type classification in the Zhengsorted dataset. Average values are recorded from five independent repetitions

Model	#Clients	Senario	Time(s)
SVM	1	Centralized	87.152
	2	Local	105.980
		Global	207.104
	5	Local	117.656
		Global	151.473
	10	Local	125.362
		Global	174.717
	20	Local	136.691
		Global	256.885
ACTINN	1	Centralized	184.950
	2	Local	176.221
		Global	452.202
	5	Local	167.839
		Global	424.889
	10	Local	169.081
		Global	512.233
	20	Local	184.533
		Global	764.668

In our detailed exploration of runtime complexities, five independent measurements were taken on the server, focusing on parameter aggregation time and communication time for sending$\backslash $ receiving model parameters. As shown in Table 3, both aggregation and communication times grow gradually with the increase in client numbers. This indicates a linear scaling in terms of both the time taken to integrate model parameters from additional clients and the communication overhead. However, potential complexities introduced at the threshold of $N=20$ lead to a deviation from a linear trend. Notably, given ACTINN’s larger parameter set, it inherently requires more time than SVM, highlighting the time complexity associated with handling more advanced models.

**Table 3:** Training aggregation and communication time comparison for cell type classification in Zhengsorted dataset. Average values are recorded from five independent repetitions

Model	#Clients	Time(s)	Aggregation time(s)	Communication time(s)
SVM	2	207.104	0.154	0.318
	5	151.473	0.275	1.063
	10	174.717	0.450	4.743
	20	256.885	1.093	21.891
ACTINN	2	452.202	1.246	1.542
	5	424.889	2.490	3.861
	10	512.233	4.483	8.317
	20	764.668	9.373	17.019

### Benchmarking of federated Geneformer for cell type identification

In this section, we benchmarked the federated implementation of Geneformer for cell type identification, contrasting its performance with SVM, ACTINN and XGBoost in the framework of scFed. The evaluation, as reported in Table 4, includes centralized, local, and global configurations using the the Zhengsorted dataset.

**Table 4:** Comparison of classification models across centralized, local and global scenarios in the Zhengsorted dataset for cell type classification. Average values and standard deviations are recorded from five independent repetitions. For runtime comparison, all models underwent 100 training iterations, while XGBoost utilized 30 boosting rounds

Scenario	Model	Client Num	Avg F1	Time(s)
Centralized	SVM	1	0.881 $\pm $ 0.00198	87.152$\pm $1.129
	ACTINN	1	0.902 $\pm $ 0.00355	184.950$\pm $2.475
	XGBoost	1	0.872 $\pm $ 0.00336	9.306$\pm $0.200
	Geneformer	1	0.905 $\pm $ 0.00162	25251.388$\pm $195.918
Local	SVM	5	0.840 $\pm $ 0.00228	117.656$\pm $0.296
	ACTINN	5	0.879 $\pm $ 0.00238	167.839$\pm $1.571
	XGBoost	5	0.841 $\pm $ 0.00112	12.027$\pm $0.415
	Geneformer	5	0.845 $\pm $ 0.00295	57718.005$\pm $240.229
Global	SVM	5	0.886 $\pm $ 0.00246	151.473$\pm $2.679
	ACTINN	5	0.902 $\pm $ 0.00270	424.889$\pm $3.998
	XGBoost	5	0.870 $\pm $ 0.00388	542.739$\pm $7.915
	Geneformer	5	0.890 $\pm $ 0.00383	57844.024$\pm $425.224

In the centralized setting, Geneformer achieved the best average F1 score of 0.905. However, in both local and global contexts, ACTINN outperformed with F1 scores of 0.879 and 0.902, respectively. Notably, Geneformer’s training duration considerably exceeded its counterparts. Even in the centralized scenario, its training time was still two orders of magnitude greater than the other evaluated models. Classification models in the Zhengsorted dataset highlighted XGBoost’s faster runtime in local and centralized setups, possibly attributed to its boosting algorithm, optimization strategy and efficient processing.

## DISCUSSION

In this study, we assessed the utility of scFed, a federated-learning-based framework, to perform cell type classification with scRNA-seq. A wide range of scenarios were simulated to assess the efficacy of scFed in handling diverse challenges associated with single-cell data analysis, including considerations for privacy preservation, robustness to dataset heterogeneity and scalability to handle massive datasets.

Within the scFed framework, we incorporated federated adaptations of advanced classification algorithms for single-cell identification, including SVM, neural network, tree-based model and Transformer-based model. In our experiments, scFed exhibited robustness in cell type classification, matching traditional centralized models while ensuring data privacy. The global models outperformed local ones through aggregating information across multiple clients.

Though Transformers are widely applied in various tasks, it is essential to recognize that the Geneformer model demands a significant training time and encompasses a large number of parameters. This raises concerns about the necessity of utilizing such a heavyweight model for this task. The trade-off between accuracy, model size and training time requires careful consideration. Exploring alternative models or methods with simpler architectures and shorter training times while retaining commendable accuracy could offer a more efficient solution for cell type classification.

Despite these promising findings, scFed does have some limitations. First, the increase in training time with the rise in client numbers could potentially limit its scalability for extremely large-scale applications. Moreover, while scFed showed robustness across different classification algorithms, the effectiveness of federated learning could be further explored with other algorithms, such as deep regression forests, or more advanced federated learning techniques. Importantly, the absence of raw data sharing does not eliminate privacy concerns. Shared model updates can inadvertently leak information, allowing potential adversaries to infer individual data attributes. Future directions should integrate scFed with privacy-preserving techniques such as trusted execution environments [30] or secure multi-party computation [31] to further strengthen scFed’s privacy guarantee.

Regarding the wider application of scFed, while its effectiveness has been demonstrated in the context of scRNA-seq analysis, its federated learning-based framework offers immense potential for other tasks in bioinformatics. Given the prevalent challenges of data privacy and the need for multi-institutional collaboration in bioinformatics, scFed’s capabilities could be leveraged to enable secure, privacy-preserving analysis across a range of biological data types.

**Key Points**

- scFed presents a unified federated learning framework for cell type identification with scRNA-seq, enabling global model building without direct raw data exchange.
- Through a unified approach, scFed facilitates benchmarking of various classifiers for cell type identification, including SVM, ACTINN, XGBoost and Geneformer, across eight datasets.
- In both intra-dataset and inter-dataset evaluations, scFed demonstrates consistent and robust performance, often matching or even slightly outperforming centralized models in terms of accuracy. Although its training duration is two to three times that of centralized models, scFed’s efficiency remains practical for cell type identification.
- Geneformer, a Transformer-based model, excels in centralized settings but shows reduced classification performance in the federated learning framework. Given its computational demands, balancing accuracy, model size and training time is essential for efficient cell type classification.

## Data Availability

The scRNAseq data are downloaded from https://doi.org/10.5281/zenodo.3357167

## CODE AVAILABILITY

The current version of scFed is implemented in python and can be found at https://github.com/digi2002/federatedSinglecell.

## Author Biographies

**Shuang Wang** is affiliated with the Institutes for Systems Genetics, West China Hospital, and is also the co-founder of Hangzhou Nuowei Information Technology Co., Ltd, Hangzhou, China.

**Bochen Shen** is a research associate at Hangzhou Nuowei Information Technology Co., Ltd, located in Hangzhou, China. His expertise includes federated learning, deep learning and bioinformatics.

**Lanting Guo** is an algorithm expert at Hangzhou Nuowei Information Technology Co., Ltd, located in Hangzhou, China. His expertise includes federated learning, deep learning and distributed computing systems.

**Mengqi Shang** is an algorithm engineer at Hangzhou Nuowei Information Technology, Hangzhou, China. Her expertise includes federated learning.

**Jinze Liu** is a professor at Department of Biostatistics, Virginia Commonwealth University, USA. Her primary research focus lies in the field of bioinformatics, specifically concentrating on the comprehensive analysis of multi-omics data to unravel intricate biological insights and patterns.

**Qi Sun** is an algorithm scientist at Hangzhou Nuowei Information Technology, Hangzhou, China. Her research interest is bioinformatics, NLP and federated learning.

**Bairong Shen** is a professor and executive director at Institutes for Systems Genetics, West China Hospital, Sichuan University. His research interest is biomedical informatics, genetics and medical systems biology.

## Appendix

scRNA-seq data exhibit significant cell-to-cell variation due to biological heterogeneity and technical effects. In our inter-dataset experiments, we conducted experiments among different datasets that included both biological and technical variables. To solely evaluate the impact of biological factors within the federated learning framework, we introduced a dataset that exhibits significant biological variability. We conducted supplementary experiments using Lee’s dataset (GEO GSE149689) [32]. Lee’s study performed scRNA-seq using PBMCs to identify factors associated with the development of severe COVID-19 infection. From this dataset, we randomly selected two samples: PBMCs from a healthy donor and a patient with COVID-19. Our comparative experiment involved two distinct cell compositions: the first, termed patient-agnostic experiment, mixed cells from both samples and distributed them across two clients; the second, referred to as the patient-specific experiment, assigned cells from each individual sample to separate clients.

As shown in Table A1, SVM’s performance is relatively stable across all scenarios, with a slight decrease in the global setting compared with the centralized one in patient-specific experiments. Contrastingly, ACTINN, XGBoost and Geneformer show greater declines in the global scenario when applied to patient-specific data. In the patient-agnostic experiments, all the models show smaller reductions in global models compared with their patient-specific counterparts, with Geneformer in particular demonstrating improved global performance compared with its patient-specific results. Our findings reveal that mixing cells from both patient samples improves global model performance, underscoring the benefit of sample diversity in our federated learning framework.

**Table A1:** Comparison of classification models between patient-specific and patient-agnostic experiments in Lee’s dataset. F1 values are reported for centralized, local and global scenarios

	Model	Senario		
		Centrialized	Local	Global
Patient-specific	SVM	0.909	0.881	0.907
	ACTINN	0.890	0.840	0.870
	XGBoost	0.893	0.861	0.865
	Geneformer	0.898	0.822	0.823
Patient-agnostic	SVM	0.909	0.904	0.908
	ACTINN	0.890	0.855	0.885
	XGBoost	0.893	0.872	0.866
	Geneformer	0.898	0.800	0.844
